# Degradation of Aflatoxin B_1_ in Moldy Maize by *Pseudomonas aeruginosa* and Safety Evaluation of the Degradation Products

**DOI:** 10.3390/foods12061217

**Published:** 2023-03-13

**Authors:** Yanhua Xu, Renyong Zhao, Chenxi Liu

**Affiliations:** College of Food Science and Engineering, Henan University of Technology, Zhengzhou 450001, China

**Keywords:** moldy maize, *Pseudomonas aeruginosa*, safety evaluation, mycotoxins, degradation aflatoxin B_1_

## Abstract

Aflatoxin B_1_ (AFB_1_) is the most harmful mycotoxin commonly found in food and feed. Pollution from AFB_1_ causes serious economic and health issues worldwide because it causes strong mutagenicity and carcinogenicity in humans and animals. In this study, *Pseudomonas aeruginosa* was used to degrade AFB_1_ in moldy maize, and the safety of this biological method was investigated using genotoxicity and cytotoxicity tests. Using response surface methodology, we established the optimal conditions for degrading AFB_1_ by the fermentation supernatant of *P. aeruginosa*. Under these conditions, the degradation rate of AFB_1_ reached 99.67%. Furthermore, the Ames mutagenicity test showed that AFB_1_ treated with *P. aeruginosa* fermentation supernatant for 72 h was not mutagenic. CCK-8 cell assay showed that AFB_1_ cytotoxicity was significantly reduced after degradation. Overall, our findings show that the fermentation supernatant of *P. aeruginosa* may be a good candidate for biodegradation of AFB_1_.

## 1. Introduction

Aflatoxins (AFs) are a class of dihydrofuran coumarin derivatives with similar chemical structures. They are mainly secondary metabolites produced by *Aspergillus flavus* and *A. parasiticus*. Other *Aspergillus* also produce aflatoxins, such as *A. noius*, *A. pseudotamarii*, and *A. fumigatus* [1,2,3,4]. AFs can easily contaminate crops and agricultural products, such as grains, feeds, seasonings, and nuts, which can widely be infected by mold under appropriate temperature and humidity conditions [5,6,7]. The global prevalence of mycotoxins detected in food crops has been estimated to be 60–80%, and this causes huge economic losses to agriculture and industry [8,9,10,11]. Corn is an important source of feed for the animal husbandry and aquaculture industries, as well as an indispensable raw material for food, medical and health care, light, and chemical industries. Corn plays a pivotal role in the development of agricultural and rural economies, with the safety of corn directly affecting the quality of livestock and poultry products, as well as human health [12]. However, owing to the characteristics of large embryos, rich nutrients, vigorous respiration, and numerous bacteria, coupled with rainfall and high temperature during the harvest period, corn is prone to mildew and germination, which facilitates its contamination by mycotoxins to varying degrees [13,14].

Currently, there are more than 600 known mycotoxins, of which Aflatoxin B_1_ (AFB_1_) is the most common and most toxic [15]. AFB_1_ is 68 times more toxic than arsenic, 10 times more toxic than potassium cyanide, and 76 times more carcinogenic than dimethynitrosamine [16]. The International Agency for Research on Cancer (IARC) classified AFB_1_ as a group I carcinogen [17]. AFB_1_ may infect food crops at various stages of growth, harvesting, transportation, and storage, thus threatening human health throughout the food chain. It has relatively stable chemical properties and is difficult to dissolve in water; however, it easily to dissolves in methanol, chloroform, and other organic solvents. It can only be destroyed at or above 280 °C [3]. The removal of AFB_1_ in a sustainable (green and efficient) manner is a current issue that urgently requires attention. To this end, microbial degradation of mycotoxins has attracted wide attention because this process does not alter the nutritional value and sensory quality of raw materials, does not produce any harmful residues, and is of low cost [18].

*Pseudomonas aeruginosa* is a gram-negative bacterium that is widely distributed in nature [19]. It requires simple nutrition for growth, has strong adaptability to the natural environment, and can produce substances that promote biodegradation [20]. It is a commonly used strain for the biodegradation of organic pollutants [21]. *P. aeruginosa* can degrade various compounds including phenols, nitrophenols, alkanes, polycyclic aromatic hydrocarbons, and aromatic compounds [22,23]. The ability of *P. aeruginosa* to degrade AFB_1_ was first reported by Sangare et al., who proved that the active components of AFB_1_ degradation are proteases [24]. Although *P. aeruginosa* can effectively degrade AFB_1_, the toxicity of the degradation products is a key issue in this degradation procedure.

Generally, carcinogenic, cytotoxic, and animal tests are conducted to detect the associated metabolites of AFB_1_. The Ames test (*Salmonella*-microsomal screening system) is a mutagenic test developed by Ames et al. that has been widely used to economically and rapidly identify the mutagenic and carcinogenic potential of chemicals [25,26,27,28]. Currently, many researchers use this test to detect the toxicity of AFB_1_ degradation products [29,30,31]. When mutants are present, nutrition-deficient bacteria revert to wild-type strains, and thus can grow visible colonies. Based on this, it can be determined whether the subject matter has no mutants [32]. The human hepatocellular carcinoma cell line, HepG2, is widely used to evaluate the cytotoxicity of various compounds. When toxic substances come into contact with cells, the survival rate of cells is reduced, and cell morphology changes [33,34,35]. The presence of AFB_1_ reportedly reduces cell survival, which may be associated with the direct impairment of specific protein function or DNA damage caused by toxins, such as protein phosphorylation in the liver or cyclic nucleotide phosphodiesterase activity [36].

We previously screened a *P. aeruginosa* M-4 strain using coumarin as the sole carbon source; the fermentation supernatant of *P. aeruginosa* M-4 had a good degradation effect on AFB_1_. Further, the supernatant retained the degradation activity following high temperature treatment, making it widely applicable [37]. In this study, the fermentation supernatant of *P. aeruginosa* was used to degrade AFB_1_ in moldy corn, and the optimal operating parameters for the degradation process were explored using a single-factor and response surface method. Ames and CCK-8 tests were used to determine the overall toxicity of AFB_1_ degradation products. We aimed to investigate the feasibility and safety of using *P. aeruginosa* to degrade AFB_1_ in moldy maize.

## 2. Materials and Methods

### 2.1. Chemicals and Reagents

Corn was purchased from the Henan Fujitai Seed Co., Ltd. (Zhengzhou, China). A *P. aeruginosa* M-4 strain was cultured in our laboratory. *Aspergillus flavus* NRRL3357 was provided by the School of Bioengineering, Henan University of Technology (Zhengzhou, China). A standard sample of AFB_1_ (2,3,6α,9α-tetrahydro-4-methoxycyclopenta[c]furo [2,3:4,5]furo [2,3-h]chromone-1,11-dione, C_17_H_12_O_6_, purity > 98%) was obtained from the Sigma Chemical Co., Ltd. (St. Louis, MO, USA). High-performance liquid chromatography-grade methanol was purchased from Fisher Scientific (Waltham, MA, USA). Biological-reagent beef extract, peptone, and agar were purchased from the Beijing Aoboxing Biotechnology Co., Ltd. (Beijing, China). Analytical grade methylene chloride, sodium chloride, and potassium dihydrogen phosphate were purchased from the Tianjin Hengxing Chemical World Manufacturing Co., Ltd. (Tianjin, China). Iodine was purchased from the Tianjin Yongda Chemical Reagent Co., Ltd. (Tianjin, China). The Ames kit was purchased from the Beijing Huizhi Heyuan Biotechnology Co., Ltd. (Beijing, China). Fetal bovine serum was purchased from Capricorn Scientific GmbH (Ebsdorfergrund, Germany). Minimum essential medium was purchased from the American Corning Co., Ltd. (Shanghai, China). The CCK-8 kit was purchased from Invigentech (San Francisco, CA, USA). HepG2 cells were purchased from iCell Bioscience, Inc. (Shanghai, China).

### 2.2. Preparation of Moldy Corn Meal

Approximately 200 g of corn grain (free of toxins) was placed in a 1000 mL glass bottle, sealed with a breathable sealing film, placed into an autoclave, and sterilized at 121 °C for 20 min. Potato dextrose agar (PDA) was inoculated with *Aspergillus flavus* NRRL3357 and incubated at 30 °C until the whole plate was covered with yellow-green mycelium. The mycelium was gently scraped with a sterile scalpel and completely rinsed with sterile distilled water to prepare 300 mL of spore suspension (1 × 10^4^ CFU/mL), which was then added to the glass bottle containing sterile corn and shaken to moisten the corn evenly. The glass bottle was sealed with a breathable sealing film and incubated statically at 28 °C. The following day, the glass bottle was shaken again to moisten the slightly dried corn grains on the top. After continuous culture for five days, the moldy corn grains were sterilized at 121 °C for 30 min to kill *A. flavus*. Following sterilization, the corn grains were transferred onto a tray, evenly paved to a thickness of approximately 2 cm, and placed in an oven set at 40 °C to dry for 2 h. After cooling to 25 °C, the corn grains were crushed using a multifunctional grinder.

### 2.3. Determination of Moisture in Corn Meal

Determination of moisture in corn meal was according to the GB/T10362-2008 “Grain and Oil inspection–corn moisture determination” as stipulated in the second drying method to determine the moisture of corn flour. Approximately 8 g (*m*_1_, accurate to 0.001 g) of corn meal was weighed in a constant weight aluminum box (*m*_0_, accurate to 0.001 g) and dried in an oven set at 130 °C (±1 °C). After 4 h, the aluminum box was covered and removed from the oven. The box was cooled to room temperature (25 °C) in a dryer and weighed (*m*_2_, accurate to 0.001 g). The formula below was used to calculate the moisture content in the corn meal as follows.

(1) $$N=\frac{m_{0}+m_{1}-m_{2}}{m_{1}}\times 100\%$$

where *N* represents the moisture in the corn meal (%), m_0_ is the mass of the aluminum box baked to a constant weight (g), *m*_1_ is the mass of the corn meal (g), and *m*_2_ represents the mass (g) of the baked sample and aluminum box.

### 2.4. Preparation of P. aeruginosa Fermentation Supernatant

*P. aeruginosa* was streaked out onto an agar plate (0.3% beef extract, 0.5% peptone, 0.5% NaCl and 1.8% agar, pH 7.0) and placed in an incubator at 37 °C for 24 h. Subsequently, three to four colonies were picked up with a sterilized inoculation ring, and transferred into 250 mL triangular bottles containing 50 mL beef extract peptone (BEP: 0.3% beef extract, 0.5% peptone, 0.5% NaCl and 1.8% agar, pH 7.0) [38]. The triangular bottle was sealed with a breathable sealing film prior to being placed in a shaker to culture for 24 h at 37 °C and 160 r/min. Following incubation, 2.5 mL culture was accurately added into a 250 mL triangular bottle containing 50 mL fresh fermentation medium (0.3% beef extract, 1% peptone, 0.1% KH_2_PO_4_, 0.85% NaCl, and 0.2% glucose, pH 7.0), sealed with a breathable sealing film, and cultured for 48 h at 37 °C and 160 r/min. After the culture, the fermentation liquid was transferred to a 50 mL centrifuge tube and centrifuged at 8000× *g* for 10 min at 4 °C, and the fermentation supernatant was stored at 4 °C until further use.

### 2.5. Optimization of the Degradation Conditions of AFB_1_ in Moldy Maize

Twenty-five grams (dry base) of moldy corn (1131 μg/kg) were weighed into a 250 mL triangular bottle, 100 mL of fermentation supernatant was added, the bottle was sealed with a breathable sealing film, and placed in a shaker to degrade for 48 h. Simultaneously, the fermentation medium of uninoculated *P. aeruginosa* was used as the blank and was combined with the moldy corn under identical conditions. The optimized factors included temperature (30, 40, 50, 60, and 70 °C), solid–liquid ratio (10, 15, 20, 25, and 30 g/100 mL), pH of the fermentation supernatant (6.2, 7.2, 8.2, 9.2, 10.2, and 11.2), and degradation time (6, 12, 18, 24, 30, 36, 42, 48, and 54 h). The AFB_1_ residue in the moldy corn was analyzed by high-performance liquid chromatography (HPLC).

Based on the results of single-factor experiments, the degradation temperature (°C), pH, and degradation time (h) were considered as independent variables (*X*_i_), and the degradation rate of AFB_1_ was considered as the response value (*Y*). Design Expert 8.0.6 software was used to create a Box–Behnken experimental design with three factors and three levels, with a total of 17 experimental runs being generated.

### 2.6. Determination of Residual AFB_1_ by HPLC

On completion of the degradation process, the triangular bottle was removed from the shaker and left to cool to 25 °C. Methylene chloride (three times the moldy corn, v/m) was then added to the triangular bottle in the ventilation cabinet, and the rotor was added and sealed with plastics wrap. The triangular bottle was placed on a magnetic agitator and stirred for 50 min. The mixture was transferred to a 100 mL centrifuge tube and centrifuged for 5 min at 5000× *g* and 4 °C twice to obtain the underlying organic phase liquid. Six milliliters of the organic phase were accurately transferred into a nitrogen blowpipe; after being dried with nitrogen at 50 °C and redissolved in a 2 mL mobile phase (methanol: water = 45:55, *v/v*), residual AFB_1_ was completely redissolved by vortex oscillation for 2 min. AFB_1_ was determined by HPLC after filtration through a 0.22 μm organic filter membrane. The system was equipped with a JADE-PAK AF-C18 column (250 mm × 4.6 mm, 5 μm) and fluorescence detector. The excitation and emission wavelengths of the detector were 360 nm and 440 nm, respectively. The mobile phase was methanol aqueous solution (methanol: water = 45:55, *v/v*) set at a flow rate of 0.8 mL/min. The analytical run time was 25 min, and the column temperature was set at 30 °C. Post-column derivatives were prepared using 0.05% iodine solution as the derivative solution. The flow rate of the derivative solution was set at 0.2 mL/min and the temperature of the derivative pool was 70 °C. A standard curve was prepared with the concentration of AFB_1_ as the abscissa and the peak area of the chromatograms as the ordinate. The AFB_1_ concentrations used in the standard curve were 1, 5, 10, 25, 50, 100, and 200 ng/mL. Consequently, the degradation rate of AFB_1_ in the moldy corn was calculated using the formula below.

(2) $$Y=\frac{X_{1}-X_{2}}{X_{1}}\times 100\%$$

where *X_1_* is the amount of residual AFB_1_ in the control sample (ng), *X_2_* is the amount of residual AFB_1_ in the test sample (ng), and *Y* is the degradation rate of AFB_1_ (%).

### 2.7. Ames Test for Mutagenicity

#### 2.7.1. Sample Preparation

*P. aeruginosa* fermentation supernatant (975 μL) and 25 μL of AFB_1_ at different concentrations (0.1, 0.5, 2.5 μg/mL) were placed in 2 mL centrifuge tubes. After vortex oscillation, they were placed in a micro-oscillator set at 50 °C and 160 r/min for 0, 1, 2 and 3 days. After the reaction, the samples were freeze-dried and dissolved in 1 mL of dimethyl sulfoxide (DMSO) to maintain the same concentration as that of the initial AFB_1_. The solvent control was DMSO, the positive control of both strains was 2-aminofluorene when S9 was added, and the positive controls of TA98 and TA100 when S9 was not added were fenaminosulf and methyl methylsulfonate, respectively.

#### 2.7.2. Determining Sample Mutagenicity

Mutagenicity was determined by plate incorporation according to the instructions of the Ames kit. Bacterial (*Salmonella typhimurium* testers strain TA98 or TA100) solution (0.1 mL) and a 0.1 mL of the sample were added to a 2.0 mL insulated top medium. S9 mixed solution (0.5 mL) was added to the metabolic activation group (0.5 mL blank S9 solution was added to the non-metabolic activation group), and was thoroughly mixed and quickly poured into the solidified bottom medium. The plate was gently rotated to evenly distribute the top medium and to lay flat for curing. Following this, the plates were transferred to an incubator and cultured inversely for 48 h at 37 °C, and the total number of colonies in each plate was counted.

### 2.8. Cytotoxicity Test

#### 2.8.1. Sample Preparation

The fermentation supernatant of *P. aeruginosa* (975 μL) and 25 μL AFB_1_ at different concentrations (1, 2.5, 5, 10, and 20 μg/mL) were placed in a 2 mL centrifuge tube. After being uniformly oscillated, they were placed in a micro-oscillator under reaction conditions of 50 °C and 160 r/min for three days to facilitate degradation. After freeze-drying, AFB_1_ was dissolved in DMSO (1%), and then diluted to its initial concentration using cell complete medium and filtered through a 0.22 μm membrane for sterilization.

#### 2.8.2. Measurement of Cell Viability

HepG2 cells at the logarithmic growth stage were counted, and the cell concentration was adjusted and then inoculated into 96-well plates at a density of 1.5 × 10^4^ cells/well. Next, the 96-well plates were placed in an incubator maintained at 37 °C and a 5% CO_2_ atmosphere for 24 h. After the culture, the medium was gently removed and washed with phosphate-buffered saline (PBS) three times. Subsequently, 100 μL of 10% CCK-8 medium was added to each well ensuring that air bubbles were not generated. The 96-well plates were placed in an incubator set at 37 °C and a 5% CO_2_ atmosphere for 2 h. The absorbance at 450 nm was measured using a microplate reader. The cell survival rate was calculated using the formula below.

(3) $$M=\frac{Experimental\;sample-Blank\;sample}{Control\;sample-Blank\;sample}\times 100\%$$

where the experimental sample contained different concentrations of cell culture medium, the control sample contained 1% DMSO of cell culture medium, and the blank sample only contained CCK-8 reagent and medium.

### 2.9. Experimental Design and Statistical Analysis

SPSS software (version 20.0) was used for variance analysis of the experimental data. Origin 9.0 software was used for mapping analysis and Design Expert 8.0.6 was used to design the response surface experiment. All experimental data are presented as the mean ± standard error of at least two measurements unless otherwise stated.

## 3. Results and Discussion

### 3.1. Degradation of AFB_1_ in Moldy Maize by P. aeruginosa Fermentation Supernatant

#### 3.1.1. Single-Factor Experiment

**Figure 1:**
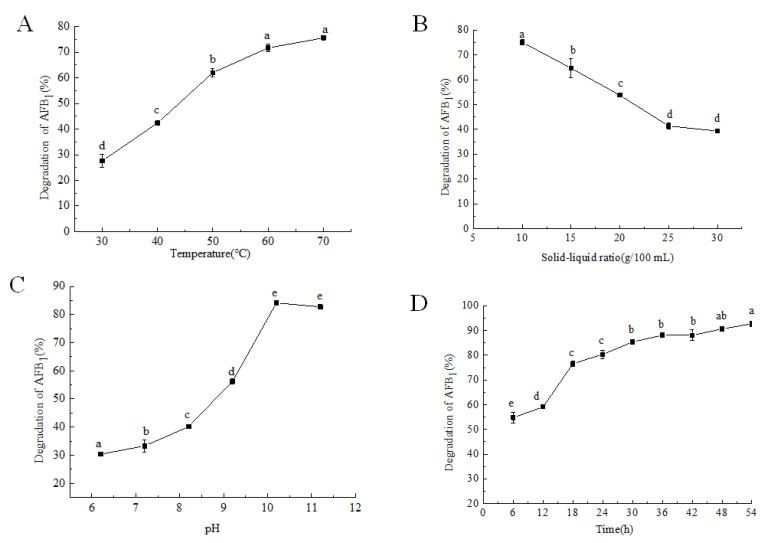
Effect of various factors on the degradation of AFB_1_ in moldy maize by fermentation supernatant. Note: (**A**–**D**) represent the effects of temperature, solid-liquid ratio, pH, and time on the degradation of AFB_1_, respectively. The different lowercase letters in each figure indicate significant differences (*p* < 0.05).

The effect of *P. aeruginosa* fermentation supernatant on AFB_1_ in moldy corn at different temperatures is shown in Figure 1A. Compared to the control sample, the degradation rate of AFB_1_ in moldy corn by the fermentation supernatant of *P. aeruginosa* increased with a temperature increase. The influence of macromolecules such as proteins and starch in moldy corn decreased the stability of AFB_1_. The activity of proteases in the fermentation supernatant was enhanced by temperature, which promoted the degradation of AFB_1_. When the temperature reached 60 °C, the degradation rate reached 71.70%, without any further significant degradation observed with an increasing temperature after this. Sangare et al. showed that the degradation rate of the AFB_1_ standard by the supernatant of *P. aeruginosa* N17-1 attained 90.2% at 55 °C, indicating that this strain additionally has the potential to tolerate a high temperature [24]. However, considering practical applications, 60 °C was selected for use in this study.

The solid–liquid ratio also had an impact on the degradation of AFB_1_ in moldy corn (Figure 1B). With an increasing solid–liquid ratio, the degradation rate of AFB_1_ decreased. There are two reasons for this trend. First, under the influence of the temperature, moldy corn meal absorbed water and expanded, turning the mixture in the triangle bottle into a paste or solid, in turn preventing the active proteases in the fermentation broth from fully being in contact with the AFB_1_ in the moldy corn. Second, the reaction system with large solid-liquid ratio has more AFB_1_ content, which will lead to the failure of AFB_1_ to degrade in time. In practical applications, both the AFB_1_ degradation rate and production efficiency should be considered; thus, a solid–liquid ratio of 25 g/100 mL was considered optimum for further experiments.

Enzyme activity is maximum only in a certain pH range; above or below this range, the activity is reduced. Based on previous research, the active component in the fermentation supernatant of *P. aeruginosa* is a protease [39]. The effects of the fermentation supernatant with different pH values on the degradation of AFB_1_ in moldy corn are shown in Figure 1C. The control group used a blank medium with the same pH value so that the effect of the alkali environment on the degradation of AFB_1_ could be excluded [40]. As depicted in Figure 1C, the degradation rate of AFB_1_ increased significantly with an increase in the pH in the range of 6.2–10.2. After a pH of 10.2, increasing the pH led to a decrease in the degradation rate, indicating that an excessively high pH value affected the activity of the degrading enzyme. Consequently, a pH of 10.2 for the fermentation supernatant of *P. aeruginosa* was selected for use in degrading AFB_1_ in moldy corn.

The degradation rate of AFB_1_ in moldy corn by the *P. aeruginosa* fermentation supernatant over time is shown in Figure 1D. In the first 18 h, the degradation rate of AFB_1_ was high, reaching more than 75% at 18 h. The degradation rate increased slowly between 18 h and 54 h. After 48 h, the degradation rate of AFB_1_ attained 90.64% and there was no significant difference between it and the degradation rate at 54 h. Thus, 48 h was selected as the optimal degradation time.

#### 3.1.2. Response Surface Optimization

**Figure 2:**
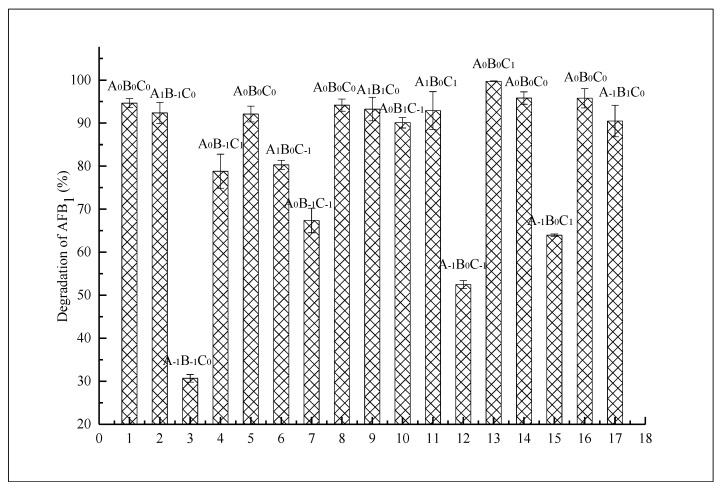
Results of response surface experiment. Note: The abscissa represents the 17 tests. In the figure, A, B, and C represent the test factors temperature, pH, and degradation time, respectively. The numbers at the lower right foot of the letter −1, 0, 1 represent the three levels of the factors, respectively.

Based on the single-factor experiments, the degradation temperature, pH, and degradation time were selected as input variables to design the factor level table of the response surface (Table 1). Design Expert 8.0.6 software was used to conduct a Box–Behnken experiment with three factors at three levels, with a total of 17 experiments. The experimental results for the response surface are presented in Figure 2. Through quadratic multiple regression fitting of the data in Figure 2, the regression model relationship was generated and is depicted in the formula below.

Y = 94.50 + 15.15A + 13.04B + 5.65C − 14.73AB + 0.28AC − 0.45BC − 14.70A^2^ − 3.11B^2^ − 7.39C^2^ (4)

**Figure 3:**
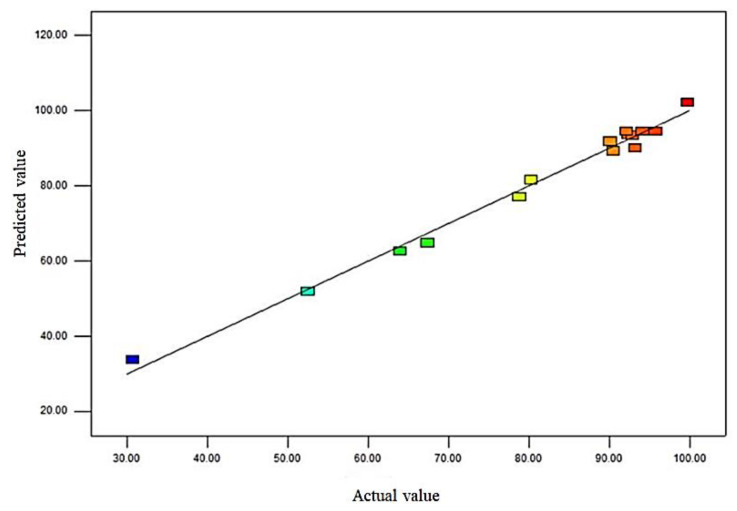
Linearity plot of the predicted and actual values. Note: 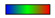 Colors ranging from blue to red indicates low to high degradation rates.

A significance test and variance analysis were conducted on the regression equation, and the results are presented in Table 2. The model *p* < 0.01 indicates that the model was very significant. The lack of fit was not significant (0.0515), indicating that the model was well fitted and that the experimental error was small. The determination coefficient R^2^ of the regression equation was 0.9904, indicating that the model could explain 99.04% of the variation in the response value [41]. The calibration determination coefficient R^2^_Adj_ = 0.9782 was close to the determination coefficient R^2^, indicating that the model had a good fitting degree and high reliability. Furthermore, there is a clear linear relationship between the predicted and actual values (Figure 3); therefore, this regression equation could be used to predict and analyze the relationship between various factors and the degradation rate of AFB_1_. The significance test showed that the primary terms A, B, and C, secondary terms A^2^ and C^2^, and interaction term AB had significant effects on the degradation rate of AFB_1_ (*p* < 0.05), whereas the secondary term B^2^, interaction terms AC and BC had no significant effects on the degradation rate of AFB_1_ (*p* > 0.05). It can be observed that the influence of all factors on the degradation rate of AFB_1_ was not a simple linear relationship, and the order of the influence of all factors was as follows: degradation temperature > pH > degradation time.

**Figure 4:**
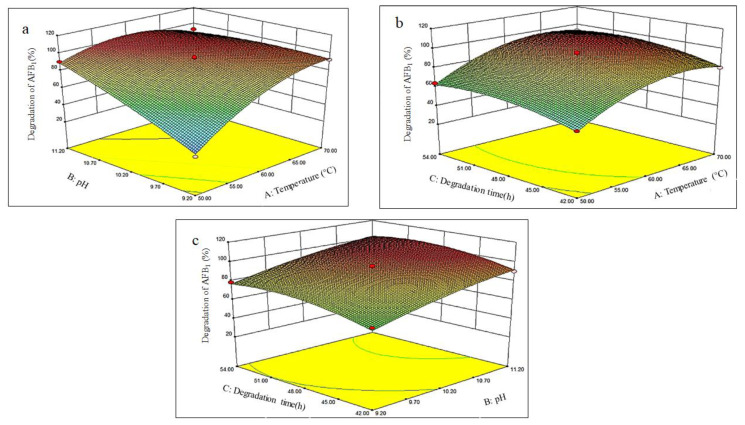
Response surface plots showing the interactive effects of various factors on the degradation rate of AFB_1._ Note: Figure (**a**) represents the effect of the interaction of the temperature and pH on the AFB_1_ degradation rate; Figure (**b**) represents the effect of the interaction of the temperature and degradation time on the AFB_1_ degradation rate; and Figure (**c**) represents the effect of the interaction of the degradation time and pH on the AFB_1_ degradation rate. 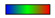 Colors ranging from blue to red indicates low to high degradation rates. The lines in the y-coordinate have no real meaning.

The influence of various factors on the degradation of AFB_1_ in moldy corn by the fermentation supernatant of *P. aeruginosa* is shown in Figure 4, and the relationships between the factors are shown on the curved surface. As observed from the regression Equation (4), the coefficients of the quadratic terms A^2^, B^2^, and C^2^ are all negative, indicating that the response surface of the quadratic polynomial has a maximum value point. The steepness of the response surface reflects the influence of the independent variable on the degradation rate of AFB_1_. The steeper the surface, the greater is the influence and vice versa. Figure 4a shows that the response surface of AB has a large slope and strong interactions, indicating that the effects of pH and temperature on the degradation of AFB_1_ would have an effect on each other. Figure 4b,c is relatively flat, indicating that there was no interaction between the effect of the degradation time on the pH and the temperature on the degradation rate of AFB_1_, which is consistent with the ANOVA results in Table 2. Design Expert software was used to optimize the experimental results, and the optimal conditions for the degradation of AFB_1_ were as follows: temperature 63.13 °C, pH 10.95, and degradation time 51.50 h. Under these conditions, the degradation rate of AFB_1_ predicted by the model was 100.5%. To perform the actual test using model predicted conditions, we set the parameters as follows: temperature 63 °C, pH 10.95, and degradation time 51.50 h. The actual degradation rate of AFB_1_ was 99.67%, which was very close to the predicted value and showed no significant difference (*p* < 0.05). This indicates that the degradation conditions optimized by this model are reliable and authentic.

### 3.2. Ames

**Figure 5:**
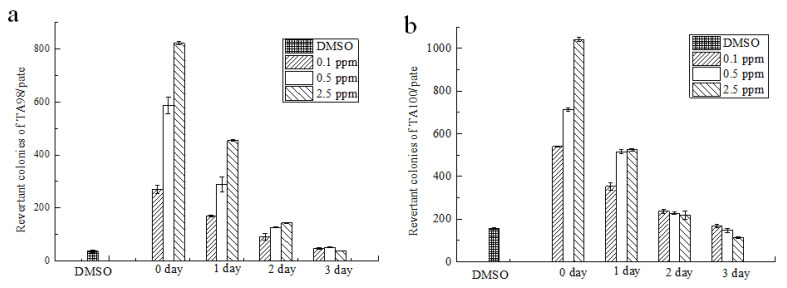
Test results of mutagenicity of AFB_1_ degradation products (−S9), Note: Figure (**a**,**b**) were mutagenicity of the degradation products of strains TA98 and TA100 after degradation for different times at initial AFB_1_ concentrations of 0.1, 0.5, and 2.5 μg/mL, respectively.

Strain TA98 was used to detect shift-code mutations while strain TA100 was used to detect base-displacement mutations. When no metabolic activators were added to activate the subjects (Figure 5), the number of revertant mutant colonies in the non-degraded group reached a maximum when the concentration of AFB_1_ was 2.5 μg/mL, and the revertant mutant colonies decreased significantly with an increase in the degradation time. After one day of degradation, the number of reverted colonies at the three different concentrations was more than twice of that in the solvent group, but there was no dose-response relationship between the concentration and the number of colonies. The results showed that the degradation products were extremely mutagenic. After two days of degradation, there was no proportional relationship between the revertant mutant colonies of the two strains. The revertant mutant colonies of TA98 and TA100 were both more than twice those of the solvent group. Although some AFB_1_ was degraded, the results indicated positive mutagenicity. After three days of degradation, the revertant mutant colonies of TA98 and TA100 were similar to those of the solvent control group, indicating that AFB_1_ degradation products had negative mutagenicity.

**Figure 6:**
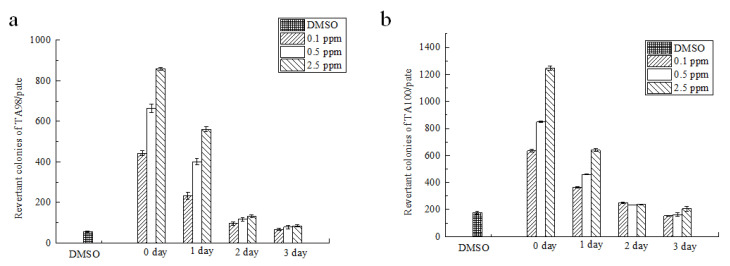
The results of mutagenicity of AFB_1_ degradation products (+S9); Note: Figure (**a**,**b**) is mutagenicity of the degradation products of strains TA98 and TA100 after degradation for different times at initial AFB_1_ concentrations of 0.1, 0.5, and 2.5 μg/mL, respectively.

Many foreign chemicals are indirect mutagenic agents that can react with DNA only if they are transformed into electron-friendly carcinogens by metabolic activation, similarly to what occurs in vivo, thus causing mutagenesis. The metabolic activation system added in the Ames test is mainly a mixed functional oxidase system, usually an S9 mixture. S9 is the supernatant obtained after the centrifugation of rat liver homogenate induced by polychlorinated biphenyls. Some cofactors, such as coenzyme II (NADP^+^), glucose-6-phosphoric acid, K^+^, Mg^2+^, and buffer, were added to S9 to form an S9 mixture. A positive result indicates that the subject is an indirect carcinogen. In the presence of S9 (Figure 6), the number of revertant mutant colonies in the untreated group was generally higher than that in the group without metabolic activators. After degradation for one day, all the tested substances were mutagenic. After two days of degradation, AFB_1_ degradation products with initial concentrations of 0.5 and 2.5 μg/mL still showed mutagenicity. After three days of degradation, the tested substances showed negative mutagenicity. Similar results were observed in Alberts’s study; the biodegradation of AFB_1_ when treated with extracellular extracts from *Rhodococcus erythropolis* coincided with a loss of mutagenicity [42]. Rao et al. proved that the cell-free supernatant of *Bacillus licheniformis* CFR1 degraded AFB_1_, and the Ames experiment showed that the mutagenicity of the degradation product disappeared [43].

### 3.3. CCK-8

**Figure 7:**
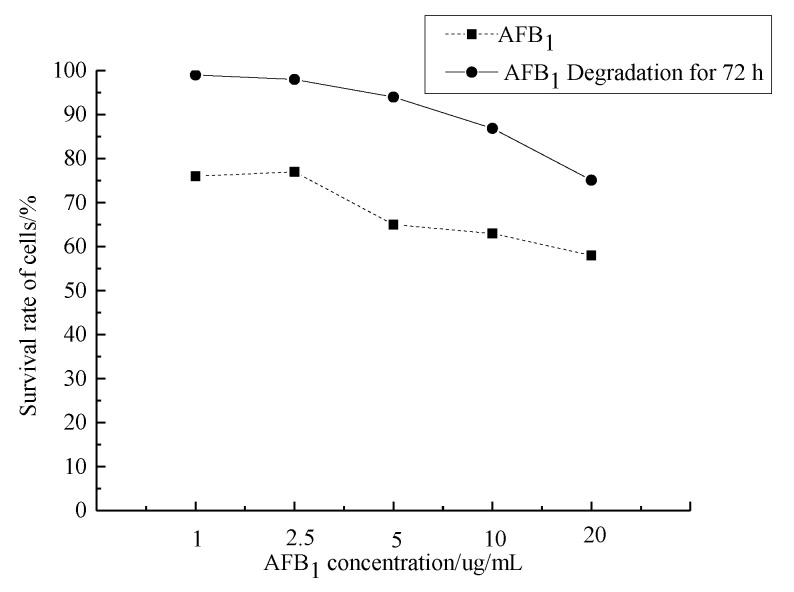
Effect of AFB_1_ and its degradation products on HepG2 viability.

The survival rate of cells decreased with increasing AFB_1_ concentration, and the toxicity of the degraded samples was significantly lower than that of AFB_1_ at the same concentration (Figure 7). When the concentration of AFB_1_ was 20 μg/mL, the cell survival rate was less than 60%. The cell inhibition rate was less than 10% when the initial concentration of AFB_1_ was less than 5 μg/mL. After the degradation of AFB_1_ at an initial concentration of 10 μg/mL, the cell survival rate was still higher than 85%. Compared with the AFB_1_ standard, the cytotoxicity of the degradation solution was significantly reduced.

**Figure 8:**
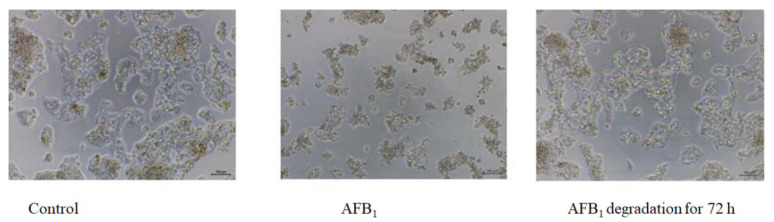
Effects of AFB_1_ and its degradation products on HepG2 morphology.

The effects of AFB_1_ and its degradation products on HepG2 cells are presented in Figure 8. HepG2 cells in the blank group were clumpy, multi-sided, and well grown. Following AFB_1_ treatment, the cell morphology changed, the surface shrank, some cells were no longer tightly grouped together, and refraction became poor. However, after AFB_1_ was degraded for 72 h, the cells still showed clumping growth, some individual cells were slightly changed, and the overall cytotoxicity was much lower than that of AFB_1_.

## 4. Conclusions

In this study, *P. aeruginosa* fermentation supernatant was used to degrade AFB_1_ in moldy corn, and the optimal degradation conditions were obtained using single-factor experiments and response surface methodology. Under the optimal conditions, the degradation rate of AFB_1_ reached 99.67%. The Ames test showed that the genetic toxicity of the degradation products was negative after 72 h of degradation. The CCK-8 assay results showed that the cytotoxicity of the degradation products decreased significantly with time. Although the Ames and CCK-8 results are generally used as preliminary assessments of AFB_1_ degradation products, they were useful in providing important information on the feasibility and safety of using *P. aeruginosa* fermentation supernatant for the degradation of AFB_1_ in moldy maize.

## Figures and Tables

**Table 1:** Variables and levels employed by the Box–Behnken.

Variables	Levels		
	−1	0	1
Temperature (A, °C)	50	60	70
pH (B)	9.2	10.2	11.2
Degradation time (C, h)	42	48	54

**Table 2:** Analysis of variance (ANOVA) of the regression equation.

Source	Sum of Squares	Freedom Degree	Variance	*F*-Value	*p*-Value	Significance
Model	5592.92	9	621.44	80.63	<0.0001	**
A	1837.09	1	1837.09	238.36	<0.0001	**
B	1361.12	1	1361.12	176.60	<0.0001	**
C	254.93	1	254.93	33.08	0.0007	**
AB	867.60	1	867.60	112.57	<0.0001	**
AC	0.32	1	0.32	0.042	0.8432	
BC	0.83	1	0.83	0.11	0.7527	
A^2^	909.82	1	909.82	118.05	<0.0001	**
B^2^	40.72	1	40.72	5.28	0.0551	
C^2^	230.09	1	230.09	29.85	0.0009	**
Residual	53.95	7	7.71			
Lack of fit	44.73	3	14.91	6.47	0.0515	
Pure error	90.22	4	2.30			
Total	5646.87	16				
R^2^ = 0.9904, R^2^_Adj_ = 0.9782						

Note: ** indicates a significant difference (*p* < 0.001).

## Data Availability

The data presented in this study are available on request from the corresponding author.
